# Adaptation of the mentor mindsets scale: validation, psychometric properties, and practical application

**DOI:** 10.3389/fpsyg.2026.1824078

**Published:** 2026-09-30

**Authors:** Burhanettin Ozdemir, Dilek Ilhan-Beyaztas

**Affiliations:** 1 College of Business, Alfaisal University, Riyadh, Saudi Arabia; 2 Department of Primary School Education, Ankara Universitesi, Ankara, Türkiye

**Keywords:** mentor mindset, growth mindset, mindset abstraction, psychometric validation, implicit theories of intelligence

## Abstract

**Introduction:**

This study aimed to adapt the Mentor Mindset Scale into Turkish and examine its psychometric properties. The scale assesses mentors’ orientations toward youth as Enforcer and Protector, conceptualizing Mentor Mindset as a pattern defined by these two dimensions.

**Methods:**

The sample comprised 316 adults (210 women, 106 men). To further examine construct distinctiveness, the Implicit Theories of Intelligence Scale was employed to test concurrent validity across fixed and growth mindset dimensions.

**Results:**

Confirmatory factor analysis supported the proposed two-factor structure, with strong comparative fit indices (CFI = 0.976, TLI = 0.955) and acceptable SRMR (0.071), although RMSEA was elevated (0.114, 90% CI [0.081, 0.150]). Factor loadings were significant, and the two dimensions were strongly correlated (*r* = 0.70). Reliability was stronger for the Enforcer subscale (α = 0.70, ω = 0.72, CR = 0.77) than for the Protector subscale (α = 0.53, ω = 0.64, CR = 0.66), indicating comparatively weaker measurement precision for the Protector dimension. Concurrent validity analyses indicated small-to-moderate negative associations with Fixed Mindset and nonsignificant associations with Growth Mindset, supporting the conceptual distinctiveness of mentor-specific orientations from general mindset beliefs. Classification analyses revealed that 47% of participants exhibited a single Mentor Mindset orientation, while 22% displayed both Enforcer and Protector tendencies. Results indicated small-to-moderate negative associations with fixed mindset and nonsignificant associations with growth mindset, supporting the cultural generalizability of mentor-specific mindset orientations while confirming their conceptual independence from general intelligence beliefs.

**Discussion:**

These findings demonstrate that the Turkish Mentor Mindset Scale is a reliable and valid measure that captures theoretically coherent constructs distinct from general mindset beliefs. The scale provides a practical tool for assessing mentor orientations and can inform the design of targeted mentoring interventions to promote adaptive mentoring behaviors in educational and developmental contexts.

## Introduction

Over the past few decades, much emphasis has been placed on psychological aspects that determine the learning process, motivation, and development patterns of people. In this list, the phenomenon of mindset has become one of the most notable theories that can be used to explain the impact that the implicit beliefs about ability, intelligence, and learning have on cognitive, emotional, and behavioral consequences under educational, as well as developmental settings (Dweck, 2006; Hong et al., 1999). In contrast to using cognitive ability as a determinant or previous performance, the mindset theory pays attention to how the belief systems of people determine how they perceive and approach challenges, how they respond to failure, and how they approach growth opportunities (Yeager and Dweck, 2012). This approach has helped to change the focus of educational and developmental research to the dynamic and flexible nature of learning as influenced by both personal ideologies and social relationships (Claro et al., 2016; Dweck and Molden, 2017).

The National Academies of Sciences, Engineering, and Medicine (2019) defines mentoring as a collaborative developmental relationship in which a more experienced or respected individual provides resources, guidance, and support that contribute to another person’s academic, social, personal, and professional development. This multidimensional view is consistent with Kram’s (1985) distinction between the career-related and psychosocial functions of mentoring. Career-related functions emphasize guidance, coaching, developmental challenge, and opportunities that support competence and professional growth, whereas psychosocial functions involve acceptance, counseling, role modeling, and support for identity and personal development. Although originally articulated within professional settings, this distinction is also relevant to educational contexts because mentoring relationships frequently combine competence-oriented guidance with interpersonal and developmental support. Evidence from higher and medical education further suggests that structured mentoring programs can contribute to students’ academic development and performance (Guhan et al., 2020; Yusof et al., 2022). Accordingly, effective mentoring extends beyond the transmission of knowledge or experience. Mentors’ beliefs, expectations, and orientations toward those they support may influence how challenge, guidance, encouragement, and protection are enacted within the mentoring relationship (Ford and Parsons, 2000; Jewell, 2007; Rowley, 1999). From this perspective, mindset theory provides a useful framework for examining the belief systems that underlie mentoring practices.

Mindset is a non-cognitive construct that denotes what people think about the kind of intelligence and ability that people possess (Dweck, 2006). According to this theoretical approach, these core beliefs influence the attitudes and behaviors of individuals connected to the process of learning, hard work, and persistence in challenging situations (Claro et al., 2016; Hong et al., 1999; Yeager and Dweck, 2012). Dweck identifies two main types of mindset; fixed mindset and growth mindset. The concept of the fixed mindset has its foundation in the idea that intelligence and capabilities are inborn and cannot be changed, whereas growth mindset is founded on the knowledge that these qualities can be acquired with the help of effort, strategies, and learning experiences (Dweck, 2000, 2016; Dweck and Molden, 2017).

It has been shown in the literature research that mindset orientations of people play a critical role in determining the learning behaviors and strategy selection (Stipek and Gralinski, 1996; Vermetten et al., 2001), levels of motivation (Haimovitz et al., 2011), and the academic achievements and achievement goal orientations (Aronson et al., 2002; Dweck, 1986; Roedel and Schraw, 1995; Blackwell et al., 2007). These results indicate that mindset is not just a reflection of the learning process but a strength of the psychological construct, which can positively influence the quality, persistence, and results of learning.

With this theoretical background, the Mentor Mindset concept provides a new angle in view of cognitive and motivational orientations of adults in supporting the development of young people. In contrast to the original model created by Dweck (2006) that centers around the beliefs that individuals hold regarding their personal abilities, Mentor Mindset helps highlight the beliefs that one holds about the potential of other people (Yeager, 2023). In that sense, the degree to which an adult trusts a young individual, in his or her ability to learn, grow, and develop, directly affects the level of self-beliefs (Ford and Parsons, 2000; Jewell, 2007), stress levels (Dziczkowski, 2013), self-esteem (Jewell, 2007), motivation (Firzly et al., 2022; Yeager, 2023), sense of belonging (Kazlauskaite et al., 2020). With this, Mentor Mindset is a relational kind of mindset that does not only inform the development of individuals, it forms the way adult individuals guide and contribute to the development of other people too.

Notably, this approach which is based on mindset is heavily determined by culture. Cultural values, social norms, and current conceptions of learning and development inform beliefs and attitudes of mentors toward the potential of the young individuals (Hofer, 2006; Ryan and Mercer, 2012; Lou and Li, 2017; Bernardo, 2021). In turn, the nature of relationships between mentors and mentees and how adults can demonstrate their faith in the potential of young individuals can be different in different cultural contexts (Obara et al., 2021; Sotto-Santiago et al., 2023). This is why Mentor Mindset should not be imagined as a universal, homogenous, and unchanging phenomenon, but as the culturally sensitive and context-specific phenomenon instead.

Although Mentor Mindset has theoretical and practical relevance, evidence concerning its measurement across cultural contexts remains limited. The Mentor Mindsets Scale developed by Yeager (2023) assesses adults’ orientations toward young people, particularly beliefs reflected in Enforcer and Protector responses to youth development and behavior. In the present study, mentoring is therefore conceptualized broadly as an adult-youth developmental relationship rather than as a specific institutional mentoring arrangement. The study does not focus exclusively on Basic Education, Higher Education, or peer mentoring, nor does it evaluate identified mentor-mentee dyads. Instead, it examines the psychometric functioning of the scale among Turkish adults as a measure of orientations that may be relevant when adults occupy guidance, instructional, caregiving, or developmental roles in relation to young people. Accordingly, potential educational applications should be interpreted as contexts for subsequent investigation rather than as evidence of validity within a particular educational level.

### Theoretical framework

Mentoring is commonly conceptualized as a developmental relationship in which a more experienced individual provides guidance and support intended to facilitate the professional and personal development of a less experienced individual (Allen et al., 2004; Kram, 1985). Kram’s (1985) framework is relevant here for defining two complementary functions of mentoring: career-related support and psychosocial support. This separation will help to grasp mentoring as a multi-dimensional developmental process rather than just as a transfer of expertise.

Career related functions include coaching, sponsorship, exposure to developmental opportunities, and provision of challenging tasks that facilitate competence and professional growth. Psychosocial functions include acceptance, counseling, friendship, role modeling and support for identity development (Kram, 1985; Ragins and Kram, 2007). These functions can be interpreted as complementary aspects of mentoring in the educational setting. Mentors can help learners through providing appropriate challenges and guidance, and also help to build confidence, belonging and personal growth (Ambrosetti and Dekkers, 2010). However, the relative emphasis of challenge, authority, support and protection can influence young people’s experience of mentoring. This functional approach offers a critical conceptual link to the Mentor Mindset model that emphasizes the beliefs and perspectives that influence adults’ understanding of youths’ abilities and what kinds of support they offer (Yeager, 2023).

Mindset Theory is the theoretical framework which best systematizes such beliefs. The theory was designed to discuss the role in which the perception of intelligence and ability contributes to learning behaviors and assumes that implicit beliefs about these attributes determine how much people are motivated, allocate efforts, and perform at school (Dweck, 2006; Yeager et al., 2013). In this context, the beliefs are usually categorized into two orientations; fixed mindset and growth mindset (Dweck, 2000).

The fixed mindset is another belief that holds that intelligence is inborn and cannot be changed (Robins and Pals, 2002). Those who support this way of thinking will be the ones more concerned with validation of performance where they look to external measures like grades or evaluations to prove capability (Dweck, 2000). This performance-based emphasis is linked to risk avoidance, less effort when faced with a difficult situation and a greater probability of disengagement when in difficulties (Dupeyrat and Mariné, 2005).

Conversely, growth mindset is an attitude that expresses the idea that intelligence and abilities could be promoted with time, appropriate strategies, and learning experiences (Dweck and Yeager, 2019). Growth mindset people tend to perceive adversities as a learning experience, see failure as a part of the development process, and continue working on long-term objectives (Claro et al., 2016; Dupeyrat and Mariné, 2005).

Even though the mindset theory was first conceived to explain the individual variation in learning and achievement opportunities, recent studies are more emphatic that the contextual and environmental factors play a crucial role in determining how mindset is formed (Bronfenbrenner, 1977). Parental beliefs, teacher mindset, and school climate have been found to be important in creating or limiting the adaptive beliefs about learning and ability (Baransi and Scharf, 2025; He and Zhang, 2024; Lou, 2025; Zhang and He, 2025). Developmental trajectories, in their turn, are seen as the result of continuous interactions with the social and educational contexts rather than just being an individual perception (Chen and Liu, 2023; Haimovitz and Dweck, 2017).

It has been empirically proved that the attitudes of teachers, parents, and mentors have a direct impact on the growth mindset, motivation, and self-efficacy of learners (Kim et al., 2024; Rattan et al., 2012; Yeager et al., 2019). This effect is especially acute in case of developmental stages including adolescence and early adulthood when outsourcing messages are particularly decisive in identity formation and long-term involvement in learning and success (Steinberg, 2008; Yeager and Dweck, 2012; Yeager et al., 2016).

In this respect, Mentor Mindset can be described as the assumptions of adults in the position of guide about the ability of young people to learn, develop, and improve. Introduced by Yeager (2023), the Mentor Mindset framework focuses on communicating high expectations and providing instructional and emotional support that is required to achieve the elevated expectations. This is not just like the conventional models of behaviorism, but it incorporates cognitive development with social emotional learning mechanisms.

The gist of Mentor Mindset is based on the notion that the potential of students is not fixed and that learning may still be attained even when it has gone wrong. In this regard, Mentor Mindset is strictly connected with the principles of the growth mindset. In comparison, there are two other orientations, Enforcer Mindset and Protector Mindset, which are conceptualized as less adaptive. Enforcer Mindset lays blame on failure to internal shortcomings like laziness or absence of discipline and it is focused on authority, compliance, and control of behavior (Yeager, 2023). This can be counterproductive to independent motivation as it will make the learning process dependent on external control (Ryan and Deci, 2000).

Protector Mindset, in turn, is an overprotective orientation, whereby adults would like to protect young people against trouble in the cause of wellbeing. Despite good intentions, this can be a restrictive style of developmental motivation because it minimizes chances of struggling and risk-taking independently and forming the ability to persevere. Overprotection is known to frustrate the need of autonomy, leading to the lack of intrinsic motivation (Ryan and Deci, 2017; Segrin et al., 2012; Schiffrin et al., 2014).

Lastly, Mentor Mindset is culturally expressed and influenced. Mentors have their expectations, guidance practices and motivational strategies shaped by cultural values (Barker, 2007). Mentoring practices could have a stronger focus on the structure and protection in collectivistic cultures and on autonomy and self-direction in individualistic cultures (Hofstede, 2001; Ryan and Deci, 2000). Such orientations of culture, also, influence the ways in which Enforcer and Protector Mindsets are exercised and involved in shaping the self-efficacy and motivation of mentees (Yeager, 2023). In its turn, it means that the effectiveness of the mentor mindset practices should be evaluated with a keen consideration of the cultural context which is vital not only in terms of the educational policy development but also in terms of the successful implementation of mentoring programs.

### The present study

Since the cultural context plays a significant role in the interpretation and implementation of mentor mindset, the issue of its effective and sound measurement emerges as a major scientific issue. Such a need makes the creation of relevant measurement tools and their cross-cultural validity rather significant. Although Mentor Mindset is a new construct in terms of operationalization, it has a strong conceptual base and is practically relevant. The main tool of measurement of this construct was created by Yeager and explained in his book 10–25: Yeager (2024). The scale is a new instrument that is meant to determine the degree to which people approve of Enforcer, Protector or Mentor Mindsets.

The scale is however limited in its applicability not only in non- English-speaking settings but also in cross-cultural comparative research due to the fact that it is only available in the English language. This is particularly limiting because such constructs as guidance, mentoring and developmental support have culturally particularized meaning and practice (Heine, 2016). By implication, it is not only desirable but also necessary methodologically to determine the validity of the Mentor Mindset Scale in cultural contexts.

The study of mentoring orientations requires measurement instruments that are both psychometrically sound and appropriate for the linguistic and cultural context in which they are applied. Accordingly, the primary objective of the present study was twofold: first, to translate and culturally adapt the Mentor Mindsets Scale into Turkish using a systematic test-adaptation procedure; and second, to evaluate the psychometric properties of the resulting Turkish version. The adaptation component addressed linguistic and conceptual equivalence across languages, whereas the psychometric component examined the internal structure, reliability, validity, and measurement invariance of the adapted instrument.

Besides the study of internal structure and reliability of the Turkish Mentor Mindset Scale, the current study clearly investigated whether mentor-specified mindset orientations are cross-culturally generalizable although they were theoretically different in relation to general beliefs about intelligence. The Implicit Theories of Intelligence Scale (Dweck, 2006) was included as an external measure to examine concurrent validity and construct distinctiveness. Existing cross-cultural studies indicate that although both growth and fixed mindsets are generally widely applicable, the patterns of their behavior and the implications on their relationships across different cultures are quite different (Heine, 2016; Bernardo, 2021). In looking at the relationships between mentor mindsets and overall intelligence beliefs, this paper gets to consider whether Mentor Mindset is a culturally flexible, role-based belief system and not a direct development of individual intelligence theories. Weak or nonsignificant relations with growth mindset and negative relationships with fixed mindset would be sufficient evidence that the phenomenon of mentor mindset orientations is culturally embedded expectations on authority, protection, and responsibility toward youth, and not universal beliefs on ability malleability (Dweck and Yeager, 2019; Yeager, 2023).

## Methodology

This study used cross-sectional survey design to test the psychometric and construct validity of the Turkish version of the Mentor Mindsets Scale. The methodological framework has involved systematic translation and cultural adaptation processes, which is succeeded by confirmatory factor analysis (CFA), reliability assessment, and validity testing. Additional analyses evaluated measurement invariance by gender and demographic differences in mindset orientations. The data were collected through an online survey site thus guaranteeing the anonymity of participants and their free involvement as per the ethics of research.

### Participants

The study sample included 316 adults (210 female, 106 male) having the average age of 40.3 years (SD 8.0). The sample was selected (Table 1) to include individuals with a range of educational levels; the biggest percentage of participants had a bachelor’s degree (63%), then graduate (23.4%), two-year university degrees (6.6%), and high-school or lower educational levels (7%). The age distribution was characterized by the largest proportion of respondents being between 41 and 50 years old (36.4%), followed by those aged 18 to 30 years (24.4%) and 31 to 40 years (23.4%). Participants aged 51 years and above represented 15.8% of the sample.

**TABLE 1:** Participant’s demographic variables.

Variable	Category/ range	Frequency	Percentage
Educational background	Two-year university	21	6.6%
	Bachelor’s degree	199	63.0%
	Graduate degree	74	23.4%
	High school or lower	22	7.0%
Age (years) Age summary	18–30	77	24.4%
	31–40	74	23.4%
	41–50	115	36.4%
	51+	50	15.8%
	–	316	100%

The validation was conducted at the level of general adult orientations toward young people rather than within a specific occupational, disciplinary, or formal mentoring population. Accordingly, inclusion was not restricted to a particular field of study or professional practice. This sampling approach was consistent with the Mentor Mindsets Scale, which assesses adults’ beliefs and orientations toward youth rather than profession-specific competencies. Field of study, occupational area, and formal mentoring experience were not collected as participant-level variables and therefore could not be examined in the present study. The sample size met the minimum CFA (Kline, 2015) requirements because the ratio of participants to items was much higher than 50:1, which is sufficient to guarantee sufficient statistical power in conducting a psychometric analysis.

### Measures

#### Mentor Mindsets Scale

The original English version of the Mentor Mindsets Scale comprises six items assessing two orientations toward youth: Enforcer Mindset (items 1–3) and Protector Mindset (items 4–6). Items 1, 2, and 3 are scored as Enforcer items, whereas items 4, 5, and 6 are scored as Protector items. Responses are recorded on a 6-point Likert scale ranging from 1 (Strongly Agree) to 6 (Strongly Disagree). None of the six Mentor Mindsets Scale items are reverse-scored. Because all items are keyed in the direction of their respective Enforcer or Protector orientation, lower item and subscale scores indicate stronger endorsement of that orientation, whereas higher scores indicate weaker endorsement. Each three-item subscale therefore ranges from 3 to 18, and a summed subscale score below 10 indicates endorsement of the corresponding orientation.

The six-item instrument was introduced in Yeager’s (2023) book rather than in a dedicated peer-reviewed psychometric validation article. Consequently, published evidence concerning the measurement properties of the original English version remains limited, which provides an additional rationale for the systematic psychometric evaluation undertaken in the present adaptation.

#### Implicit Theories of Intelligence Scale (Mindsets Scale)

To assess concurrent validity, the 8-item Implicit Theories of Intelligence Scale (Dweck, 2006) was used. Fixed Mindset was represented by items 1, 2, 4, and 6, whereas Growth Mindset was represented by items 3, 5, 7, and 8. Responses were recorded on the same 6-point Likert scale. Before computing the scale scores, items 1, 2, 4, and 6 were reverse-scored using the transformation (7-x), such that response categories 1 through 6 were recoded as 6 through 1, respectively. Items 3, 5, 7, and 8 retained their original coding. The resulting Fixed Mindset and Growth Mindset scores were used in the concurrent validity analyses.

### Translation and adaptation procedure

The Mentor Mindsets Scale was translated and culturally adapted in accordance with the International Test Commission guidelines for test adaptation (International Test Commission, 2017). The procedure comprised five sequential stages. First, two bilingual specialists in educational psychology translated the original English items into Turkish. Second, the resulting Turkish translations were compared and aligned to establish a common version. Third, this version was back-translated into English by a native English speaker who had not been provided with the original wording of the scale. Fourth, an expert panel evaluated the translated version with respect to semantic, idiomatic, and conceptual equivalence between the English and Turkish forms. Fifth, cognitive interviews were conducted with five adults to examine item comprehensibility and cultural suitability. The Turkish form resulting from these stages was subsequently used for data collection and psychometric evaluation.

### Data collection

Data were collected in Türkiye through an online survey distributed via institutional email and social media. All participants provided informed consent before completing the survey. The study protocol was approved by the relevant ethics committee of Erzincan Binali Yıldırım University (approval no. E-88012460-050.04-495271). The missing data was minimal and less than 2%. The missing data were dealt with through full information maximum likelihood (FIML) estimation in structural equation modeling.

### Data analysis

#### Confirmatory factor analysis

The lavaan package (version 0.6-19; Rosseel, 2012) in R (version 4.x) was used to conduct confirmatory factor analysis (CFA). A confirmatory rather than exploratory approach was selected because the present study concerned the cross-cultural adaptation of an existing instrument with an explicitly specified two-factor structure, rather than the development of a new scale or the identification of an unknown dimensional structure. Accordingly, the primary structural question was whether the proposed Enforcer and Protector configuration could be reproduced in the Turkish version. Given the ordinal Likert-type responses, the weighted least squares mean, and variance adjusted (WLSMV) estimator with polychoric correlations was used (Li, 2016). The model specified Enforcer (items 1–3) and Protector (items 4–6) as correlated latent variables. Model fit was evaluated using the chi-square statistic, comparative fit index (CFI), Tucker-Lewis index (TLI), root mean square error of approximation (RMSEA), and standardized root mean square residual (SRMR; Hu and Bentler, 1999).

#### Reliability analysis

Cronbach’s alpha (α) and McDonald’s omega (ω) were used to evaluate internal consistency (Sijtsma, 2009). Composite reliability (CR) and average variance extracted (AVE) were additionally calculated from the standardized CFA loadings. CR was calculated as (Σλ)^2^/[(Σλ)^2^ + Σ(1 - λ^2^)], and AVE as the mean of the squared standardized loadings. Values of CR ≥ 0.70 and AVE ≥ 0.50 were used as reference criteria (Hair et al., 2019; Malhotra and Dash, 2011). McDonald’s omega was estimated separately from the CFA-based CR calculation and was therefore treated as a complementary reliability coefficient rather than assumed to be numerically identical to CR.

#### Validity analysis

Discriminant validity was evaluated using the Fornell-Larcker criterion and the heterotrait-monotrait ratio (HTMT; Henseler et al., 2015). For the Fornell-Larcker assessment, the square root of AVE for each factor was compared with the standardized latent correlation between Enforcer and Protector. HTMT was calculated from the model-implied item correlations derived from the standardized WLSMV CFA solution rather than from the Pearson correlations reported in the descriptive item correlation matrix. Specifically, within-factor indicator correlations were obtained as the products of their standardized loadings, whereas between-factor indicator correlations were obtained as the products of the corresponding standardized loadings and the latent factor correlation. The HTMT coefficient was then calculated as the mean absolute between-factor item correlation divided by the geometric mean of the mean absolute within-factor item correlations. A value below 0.85 was used as the reference criterion.

#### Mindset classification

The original scoring protocol was used to classify participants based on the summed Enforcer and Protector subscale scores. A score below 10 indicated endorsement of the corresponding orientation. Participants scoring below 10 only on the Enforcer subscale were classified as Predominantly Enforcer, whereas those scoring below 10 only on the Protector subscale were classified as Predominantly Protector. Participants scoring below 10 on both subscales were classified as Dual Mindset, and those scoring 10 or above on both subscales were classified as Mentor Mindset.

#### Group differences

Chi-square tests were used to examine associations between mindset categories and demographic characteristics, including gender and educational background.

#### Measurement invariance

Multi-group CFA was used to test measurement invariance among genders sequentially with constraint models: configural (equal form), metric (equal loadings), scalar (equal intercepts), strict (equal residuals), and structural (equal means). Scaled chi-squared difference tests (Satorra-Bentler; Satorra and Bentler, 2001) and fit index changes (0.010–0.015; Chen, 2007) were used to compare models.

## Results

The results are presented according to the two principal components of the adaptation study. The first section describes the outcome of the translation and cultural adaptation procedure, followed by the psychometric evaluation of the Turkish version through preliminary analyses, confirmatory factor analysis, reliability and validity assessment, classification analyses, demographic comparisons, and measurement invariance testing.

### Translation and cultural adaptation

The Turkish version of the Mentor Mindsets Scale was developed through the sequential translation and cultural adaptation procedure described in the Methodology section. Two bilingual specialists first produced Turkish translations of the six original items, and these versions were subsequently compared and aligned into a common Turkish form. The aligned version was then back-translated into English by a native English speaker who had not been provided with the original item wording. This step enabled comparison of the translated content with the conceptual meaning represented in the source instrument.

The resulting Turkish version was subsequently evaluated by an expert panel with specific attention to semantic, idiomatic, and conceptual equivalence. Cognitive interviews with five adults constituted the final stage of the adaptation process and were used to examine whether the translated items were understandable and culturally appropriate for Turkish respondents. Completion of these sequential procedures produced the Turkish form that was administered to the study sample and subjected to the psychometric analyses reported in the following sections.

### Preliminary analyses

Table 2 provides descriptive statistics and bivariate correlations of all study variables; mean item scores became between 2.52 (item 5) and 4.91 (item 6) with standard deviations of 0.60–1.52, which is a satisfactory item variance. The values of skewness were within the range of -0.85 to 1.24 and kurtosis values had a range of -0.92 to 1.87 indicating there were no extreme diversion from normality. The rate of missing data was negligibly small (<2%) and missing data were addressed through FIML estimation.

**TABLE 2:** Descriptive statistics and correlations among study variables.

Variable	*M*	*SD*	1	2	3	4	5	6	7	8
Item 1	3.25	1.52	–							
Item 2	3.21	1.44	0.64*	–						
Item 3	4.39	1.52	0.46**	0.47**	–					
Item 4	2.98	1.29	0.56**	0.51**	0.32**	–				
Item 5	2.52	1.07	0.45**	0.44**	0.24**	0.51**	–			
Item 6	4.91	1.43	0.27**	0.24**	0.23**	0.34**	0.23**	–		
Enforcer	10.86	3.46	0.87**	0.88**	0.75**	0.58**	0.49**	0.31**	–	
Protector	10.41	2.59	0.54**	0.50**	0.32**	0.87**	0.73**	0.69**	0.53**	–

*N* = 316. *p < 0.01, **p < 0.001. Correlations in this table are Pearson correlations among observed item and composite scores. The Enforcer-Protector correlation at the latent level is reported separately in the CFA results.

### Confirmatory factor analysis

The WLSMV estimator indicated that the correlated two-factor model provided generally acceptable fit to the data, χ^2^(8) = 40.81, *p* < 0.001; CFI = 0.976; TLI = 0.955; RMSEA = 0.114, 90% CI [0.081, 0.150]; SRMR = 0.071. Although the RMSEA exceeded the conventional 0.08 criterion, this index should be interpreted cautiously in the present model because the model has only eight degrees of freedom. RMSEA is known to perform less reliably in models with few degrees of freedom and may yield inflated estimates of model misfit under such conditions (Kenny et al., 2015). In contrast, the CFI (0.976) and TLI (0.955) indicated strong comparative fit, and the SRMR (0.071) remained within the conventional acceptable range. Given the low degrees of freedom and the convergence of the remaining fit indices, the elevated RMSEA was interpreted as a qualification to, rather than sufficient evidence against, the adequacy of the proposed two-factor structure.

Table 3 shows standardized factor loadings. The standardized loadings of the items varied on their respective factors significantly (*p* < 0.001) and ranged between 0.395 (item 6) to 0.868 (item 4) on the Protector factor and between 0.572 (item 3) to 0.810 (item 2) on the Enforcer factor. Enforcer and Protector factors had a strong correlation (*r* = 0.696, *p* < 0.001) showing related, but different constructs.

**TABLE 3:** Standardized factor loadings for the mentor mindsets scale.

Item	Content	Enforcer	Protector
mm1	Young people fail because they are too lazy	0.790**	–
mm2	Young people who struggle lack work ethic	0.810**	–
mm3	Most important values are obedience and respect	0.572**	–
mm4	Young people are fragile and give up easily	–	0.868**
mm5	Failure debilitates performance	–	0.592**
mm6	Better to let only knowledgeable youth speak	–	0.395**

All loadings significant at **p < 0.001. WLSMV estimator with polychoric correlations. Interfactor correlation: *r* = 0.696.

Figure 1 depicts the path diagram that represents a two-factor confirmatory factor analysis (CFA) model standardized estimates of the Turkish adaptation of the Mentor Mindsets Scale. Two latent constructs are Enforcer and Protector which are measured by three observed items each. Items 1–3 indicate the Enforcer factor with factor loading standardized between 0.57 and 0.81, and Items 4–6 indicate the Protector factor which is standardized between 0.40 and 0.87. There is a positive and significant correlation between the two latent factors (*r* = 0.70, *p* < 0.001), implying that there are different but related dimensions of mentor mindsets. In general, the tendency in terms of loadings favors the suggested two-factor scale organization.

**FIGURE 1:**
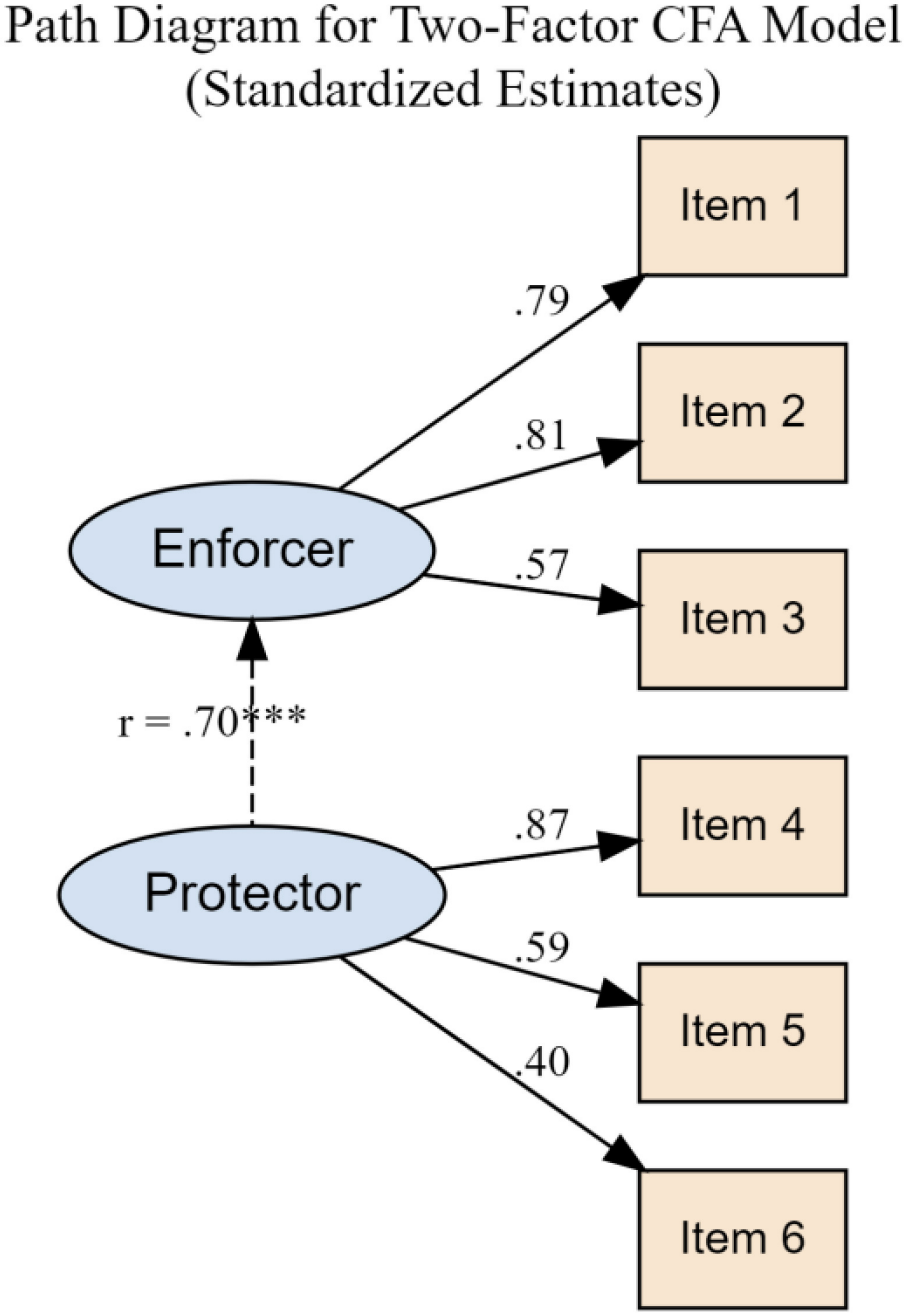
Two-factor CFA model path diagram.

### Reliability and convergent validity

Table 4 presents internal consistency and convergent validity estimates. Cronbach’s alpha was 0.70 for the Enforcer subscale and 0.53 for the Protector subscale, while McDonald’s omega was 0.72 and 0.64, respectively. Recalculation of composite reliability from the standardized CFA loadings yielded CR = 0.77 for Enforcer and CR = 0.66 for Protector. The AVE values were 0.54 and 0.42, respectively. Thus, the Enforcer factor met the reference criteria for both CR and AVE, whereas the Protector factor fell below the recommended thresholds for both indices.

**TABLE 4:** Reliability and convergent validity indices.

Factor	Items	α	ω	CR	AVE
Enforcer	3	0.70	0.72	0.77	0.54
Protector	3	0.53	0.64	0.66	0.42

α, Cronbach’s alpha; ω, McDonald’s omega; CR, composite reliability; AVE, average variance extracted.

Item mm6 (“Better to let only knowledgeable youth speak”) also demonstrated a comparatively low standardized loading (0.395). It was retained to preserve correspondence with the original six-item instrument and because removing one of only three Protector items would further restrict the content coverage of this brief subscale. Nevertheless, its relatively weak loading supports the need to examine the wording and conceptual representation of this item in future refinement studies. Combined with its relatively low alpha and omega coefficients, these results indicate weaker reliability and convergent validity for the Protector dimension and warrant caution when interpreting scores from this three-item subscale.

### Discriminant validity

The squared correlation coefficient between Enforcer and Protector factors was 0.484 (0.696^2^), and this was less than the AVE of Enforcer, but more than the AVE of Protector (0.42). This trend indicates that the Enforcer factor has sufficient discriminant validity, but the Protector factor has relatively low discriminant validity. The HTMT ratio between factors was 0.72, below the conservative 0.85 threshold (Henseler et al., 2015), supporting discriminant validity.

### Concurrent Validity with Growth Scale

Concurrent validity of the Mentor Mindset Scale was examined by correlating its two dimensions (Enforcer and Protector) with the two-factor Mindset Scale, comprising Fixed Mindset and Growth Mindset dimensions. Confirmatory factor analysis supported the adequacy of the two-factor model for the Mindset Scale. The model demonstrated acceptable to good fit, χ^2^(19) = 69.114, CFI = 0.958, TLI = 0.938, RMSEA = 0.091. Although the RMSEA value exceeded the conventional cutoff, the CFI and TLI indices indicated good comparative fit, supporting the use of the two-factor structure for validity analyses.

Pearson correlations between total scores are presented in Table 5. The Enforcer (*r* = -0.20, *p* < 0.001) and Protector (*r* = -0.30, *p* < 0.001) scores were negatively associated with Fixed Mindset. Importantly, because lower Enforcer and Protector scores represent stronger endorsement of these orientations, the negative coefficients indicate that higher Fixed Mindset scores were associated with stronger endorsement of Enforcer and Protector orientations. Thus, the negative numerical sign should not be interpreted as indicating a more adaptive mentoring orientation. In contrast, the nonsignificant associations of Enforcer (*r* = 0.04, *p* = 0.47) and Protector (*r* = 0.04, *p* = 0.46) with Growth Mindset indicate little evidence of a systematic relationship between these constructs.

**TABLE 5:** Pearson correlations between mentor mindset dimensions and growth-fixed mindset.

Variable	1	2	3	4
1. Enforcer	–			
2. Protector	0.53**	–		
3. Fixed mindset	-0.20**	-0.30**	–	
4. Growth mindset	0.04	0.04	-0.54**	–

Values represent Pearson correlations among observed composite scores. The CFA-based latent correlation between Enforcer and Protector was *r* = 0.696. **p < 0.001.

Fixed Mindset and Growth Mindset were strongly and negatively correlated (*r* = -0.54, *p* < 0.001). The direction of this association is consistent with mindset theory, in which fixed and growth orientations represent theoretically opposing beliefs regarding the malleability of intelligence and ability (Dweck, 2006; Dweck and Yeager, 2019). The corrected correlation therefore supports the expected distinction between the two mindset dimensions.

### Mindset classification

The cutoff was retained from the original scoring protocol and was not empirically optimized using the present Turkish sample. Based on the set cutoff (sum score below 10-endorsed), the respondents were divided into four mindset categories (see Figure 2). The results were: Mentor Mindset (*n* = 148, 46.8%), Dual Mindset (*n* = 70, 22.2%), Predominantly Protector (*n* = 53, 16.8%), Predominantly Enforcer (*n* = 45, 14.2%). Almost 50 % of the sample proved to have an equal Mentor orientation and about one-quarter of them had both Enforcer and Protector tendencies.

**FIGURE 2:**
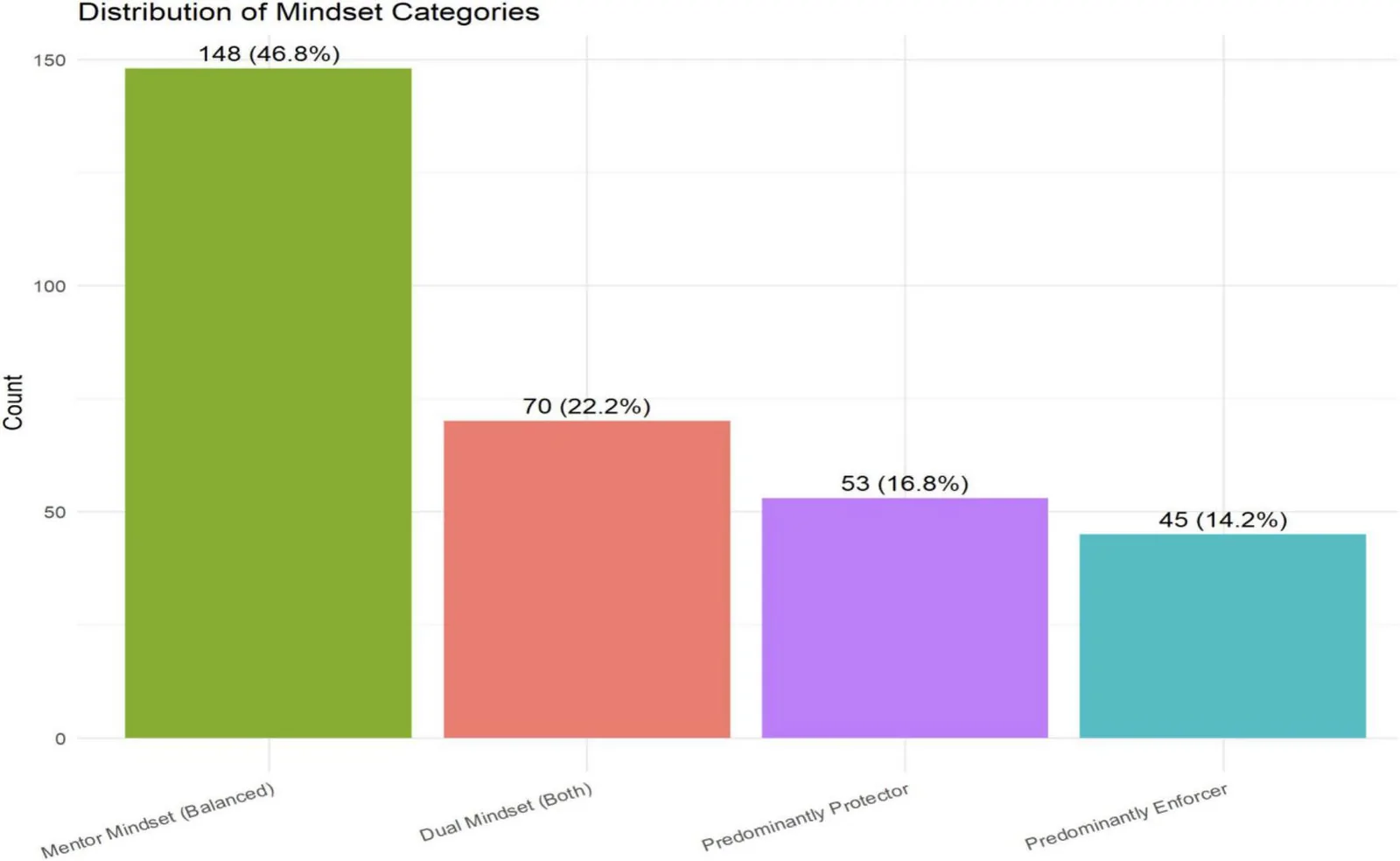
Distribution of mindset categories.

### Group differences

Chi-square tests were used to examine associations between mindset categories and demographic characteristics. There was no significant association between mindset category and gender, χ^2^(3) = 3.37, *p* = 0.338. Mindset category was significantly associated with educational background, χ^2^(9) = 21.88, *p* = 0.009, Cramér’s V = 0.15, indicating a small association. Given the cross-sectional design, this finding should not be interpreted as evidence that educational background causes differences in mindset orientation.

### Measurement invariance across gender

Multi-group CFA tested measurement invariance across gender (Table 6). The configural model (equal form) demonstrated acceptable fit: χ^2^(16) = 41.22, *p* = 0.001; CFI = 0.940; RMSEA = 0.095. Sequential constraint models showed that metric invariance (equal loadings: Δχ^2^ = 1.00, Δ*df* = 4, *p* = 0.910; ΔCFI = 0.008), scalar invariance (equal intercepts: Δχ^2^ = 7.13, Δ*df* = 4, *p* = 0.129; ΔCFI = 0.008), strict invariance (equal residuals: Δχ^2^ = 7.88, Δ*df* = 6, *p* = 0.247; ΔCFI = 0.004), and structural invariance (equal means: Δχ^2^ = 0.72, Δ*df* = 2, *p* = 0.699; ΔCFI = 0.003) were all supported. Changes in CFI and RMSEA were minimal, well below critical thresholds (ΔCFI < 0.010, ΔRMSEA < 0.015; Chen, 2007). These findings provide support for measurement invariance across gender according to the applied change-in-fit criteria. However, the results should be interpreted cautiously given the smaller male subgroup and the comparatively elevated RMSEA of the configural model.

**TABLE 6:** Measurement invariance tests across gender.

Model	χ ^2^	*Df*	CFI	RMSEA	Δχ^2^	Δ *df*	*P*	Δ CFI	Δ RMSEA
Configural	41.22	16	0.940	0.095	–	–	–	–	–
Metric	42.31	20	0.948	0.079	1.00	4	0.910	0.008	-0.016
Scalar	49.37	24	0.940	0.077	7.13	4	0.129	0.008	-0.001
Strict	58.28	30	0.935	0.072	7.88	6	0.247	0.004	-0.006
Structural	59.03	32	0.938	0.068	0.72	2	0.699	0.003	-0.004

Scaled chi-square difference tests using Satorra-Bentler correction. CFI, comparative fit index; RMSEA, root mean square error of approximation.

## Discussion

One of the main objectives of this study is to examine the psychometric validity and reliability of the Turkish adaptation of the Mentor Mindsets Scale, which measures Enforcer and Protector orientations toward young people, taking into account the cultural context. The findings showed the reliability, validity, and structural integrity of the scale. The two-factor structure was found to be acceptable with confirmatory factor analysis (CFI = 0.976, TLI = 0.955, SRMR = 0.071) though the RMSEA (0.114) was a little bit higher than desired, probably because the model has only a few items. Factor loadings were all significant and there was a strong relationship between factors (*r* = 0.70) which indicated related but different constructs.

Reliability evidence was stronger for the Enforcer dimension than for the Protector dimension. The Enforcer subscale demonstrated acceptable internal consistency and composite reliability (α = 0.70, ω = 0.72, CR = 0.77), whereas the Protector subscale produced lower estimates (α = 0.53, ω = 0.64, CR = 0.66). The Protector AVE was also below the recommended 0.50 criterion (AVE = 0.42). Although the three-item length of the subscale may contribute to lower reliability estimates, these findings indicate that the Protector dimension does not currently demonstrate the same level of measurement precision as the Enforcer dimension. Protector scores should therefore be interpreted cautiously, and further research should examine whether refinement or expansion of its item content improves reliability and convergent validity.

Evidence concerning the discriminant validity of the Enforcer and Protector dimensions was more nuanced. The Fornell-Larcker assessment confirmed the discriminant validity of the Enforcer factor, but showed, comparatively, less differentiation on the Protector factor, with the square root of the Protector AVE (0.648) being smaller than the latent inter-factor correlation (0.696). In contrast, the HTMT coefficient derived from the model-implied correlation structure of the standardized WLSMV CFA was 0.72, below the 0.85 reference criterion. These findings suggest that evidence for the discriminant validity is not consistent across the criteria. More specifically, the Protector dimension has a wider overlap with Enforcer and thus needs to be interpreted with caution. Replication across independent samples and additional investigation of the Protector item content is warranted to determine the stability of the difference between the two dimensions across populations.

The second objective of this study was to examine the relationship between general mindset theory, which concerns beliefs about the malleability of intelligence and ability, and the Enforcer and Protector orientations defined within the Mentor Mindsets framework. There was not a significant relationship between either the Protector or the Enforcer dimension and Growth Mindset, indicating that adults’ orientations toward young people are conceptually different from overall beliefs about ability development. This distinction aligns with Dweck and Yeager’s (2019) understanding that mindset beliefs are situated in specific social and developmental environments and with Yeager’s (2023) conception of Mentor Mindset as an interpretation and response to the potential of young people by adults. The negative relations of both dimensions with Fixed Mindset also indicate some theoretical relationship to general ability beliefs, though they are small in magnitude, so the constructs are not the same. This interpretation also aligns with evidence indicating that beliefs from parents, teachers, and others who are important to youth are embedded within the social environment in which they are interpreting ability and development (Haimovitz and Dweck, 2017; Yeager et al., 2019). The results thus suggest that Mentor Mindset is not a simple extension of the growth-fixed mindset continuum, but rather a relational and role-based phenomenon, driven by beliefs about guidance, responsibility and youth development. The cross-sectional design, however, does not allow for the drawing of causal inferences about these relationships.

The classification analysis revealed that 47% of respondents had a balanced Mentor Mindset and 22% were both Enforcer and Protector. Educational background was associated with the distribution of mindset categories; however, this finding is descriptive and does not establish that education or professional training influences mentor orientations. All these orientations are congruent with Kram’s (1985) conceptualization of mentoring as a multidimensional developmental relationship of challenge and psychosocial support. It also implies that adults’ reactions to youth might not be determined by a single orientation from situation to situation. Culturally, the expectations around authority, autonomy, guidance, and protection may differ based on broader social and relational norms (Barker, 2007; Hofstede, 2001) highlighting the need to examine the Mentor Mindsets construct in various cultural contexts.

The distinction between Enforcer and Protector orientations may also be considered in relation to self-determination theory. Ryan and Deci (2000) 2017 emphasize that adaptive developmental environments are ones that provide support for autonomy while offering the right structure and interpersonal support. In this view, excessive focus on control may limit autonomy and excess focus on protection may limit opportunities for independent engagement with challenge. While these theoretical links are important in explaining the Enforcer and Protector dimensions, the focus in the present study was on adults’ beliefs, not observed mentoring behaviors and youth outcomes. Further research is therefore needed to determine how these orientations are expressed behaviorally within actual adult-youth relationships.

All in all, the Turkish Mentor Mindsets Scale is an efficient tool to assess the implicit beliefs of adults in relation to children. Its conciseness makes it useful in large-scale or applied contexts, and its achieved gender invariance makes it possible to make meaningful group comparisons. Theoretically, the findings indicate that the Mentor Mindset does not reflect the absence of Enforcer and Protector orientations, but rather their balanced and context-sensitive structuring. While the Mentor Mindset is related to general mindset beliefs, it is conceptually distinct from them, as it reflects role- and responsibility-based approaches in adult–youth relationships rather than beliefs about the malleability of individual abilities. In this respect, the Mentor Mindset provides a strong theoretical foundation for interventions aimed at fostering supportive and development-oriented adult–youth relationships.

### Limitations and future directions

Several limitations should be considered when interpreting the findings. The cross-sectional design precludes causal inference and does not permit evaluation of the stability or change of mentor orientations over time. The exclusive use of self-report measures may also introduce social desirability or response biases, supporting the inclusion of behavioral, observational, or multi-informant measures in future research. In addition, the present validation was conducted with an adult sample rather than within identified mentor-mentee dyads or a specific mentoring program. The study therefore does not establish whether the scale functions equivalently across Basic Education, Higher Education, parenting, institutional mentoring, or other adult-youth developmental settings, and it should not be interpreted as evidence specific to peer mentoring. Future studies should evaluate the instrument separately within clearly defined populations, including teachers at different educational levels, parents, formal mentors, and community-based mentors, and examine whether scale scores are associated with observed mentoring practices and mentee outcomes.

The absence of detailed information concerning participants’ occupational fields, disciplinary backgrounds, and formal mentoring experience also limits the extent to which the present findings can be generalized to specific professional or mentoring populations. Future validation studies should include these characteristics explicitly and examine the scale across clearly defined groups, such as teachers, parents, formal mentors, and professionals who regularly work with young people.

Even though most of the fit indices were satisfactory, the high value of the RMSEA indicates that the model can be improved or the number of items can be increased especially to improve the reliability of the Protector subscale. The low relationship between Enforcer orientation and fixed-mindset beliefs also requires further investigation with the help of the similar concepts, like the type of attribution, self-efficacy, and mentoring behaviors. Intervention and cross-cultural research may help to define the role of culture and determine the possibility to alter mindset orientations with the help of professional training or special training.

The structural analysis was confirmatory and tested the two-factor configuration specified by the original instrument. An exploratory factor analysis on an independent subsample and formal comparisons with plausible alternative structures were not conducted. Although this approach was consistent with the objective of evaluating whether an established theoretical structure was retained after cross-cultural adaptation, it limits the extent to which alternative dimensional representations can be ruled out. Future studies should therefore replicate the Turkish version in independent samples and compare the proposed model with plausible competing structures, including exploratory solutions where appropriate.

Future studies should examine criterion-related and predictive validity using variables external to the Mentor Mindsets scoring system, such as observed mentoring behaviors, mentoring-related attitudes, or relevant developmental outcomes. Because classification depends on the specified cutoff, the reported category proportions should be regarded as threshold-dependent descriptive results, and future studies should examine their robustness across alternative thresholds.

## Conclusion

The study provides psychometric evidence concerning the Turkish adaptation of the Mentor Mindsets Scale and its proposed Enforcer and Protector dimensions. The two-factor structure was supported by confirmatory factor analysis, although the distinction between the dimensions was less clear for the Protector factor. Reliability and convergent validity were also stronger for Enforcer than for Protector, indicating that Protector scores should be interpreted cautiously and further evaluated in independent studies. The relatively low internal consistency of the Protector subscale indicates that its scores should be interpreted cautiously and further evaluated in future studies. The scale had evidence supporting measurement invariance between the sexes and was useful in categorizing the participants into theoretically meaningful categories of mindset. Approximately half of the sample portrayed an equal Mentor Mindset, and around a quarter supported both restricting orientations, which is reflective of salient targets of intervention.

The Turkish Mentor Mindsets Scale provides researchers and practitioners an instrument to evaluate adult beliefs regarding youth that could facilitate or limit effective mentorship. The tool allows making reliable judgments about the intervention requirements by distinguishing between enforcement-oriented and protection-oriented belief systems. Future studies are needed to explore how the scale predicts mentoring behaviors and youth outcomes, explore cultural differences in mindset distributions, and create interventions based on evidence to help change restrictive mindsets to balanced Mentor orientations.

Since the educational system is becoming more and more aware of the significance of supportive adult-youth relations, the instruments that can measure the belief systems behind these relationships become essential. The Turkish Mentor Mindsets Scale is an important inclusion to the measurement instrument, as it allows one to investigate what contributes to successful mentorship and assesses interventions aimed at fostering adult abilities to contribute to youth development by providing proper challenge, high standards, and sincere belief in the ability of young people to grow.

## Data Availability

The raw data supporting the conclusions of this article will be made available by the authors upon reasonable request to the corresponding author, without undue reservation.
